# Cryometamorphosis expression patterns of fruiting body development in the mushroom-forming fungus *Armillaria ostoyae*

**DOI:** 10.3897/imafungus.17.186942

**Published:** 2026-08-06

**Authors:** Ji-Jiang Zhou, Yi-Jing Zhao, Qi Liu, Jin Liu, Si-Hong Li, Zhong-Yi Wang, Zhao-Xiang Zhu, Xin-Shuai Xiong, Rui-Qing Ji

**Affiliations:** 1 Engineering Research Center of Edible and Medicinal Fungi, Ministry of Education, Jilin Agricultural University, Changchun 130118, China College of Resources and Environment, Huazhong Agricultural University Wuhan China; 2 College of Resources and Environment, Huazhong Agricultural University, Wuhan 430070, China Engineering Research Center of Edible and Medicinal Fungi, Ministry of Education, Jilin Agricultural University Changchun China

**Keywords:** *Armillaria ostoyae*, Cryometamorphosis, Developmental priming, Genetic transformation, Low-temperature induction, Zn(II)_2_Cys_6_ transcription factor

## Abstract

*Armillaria ostoyae* is an economically important forest pathogen whose reproductive development is regulated by environmental cues. Although low temperature is recognized as an important morphogenetic stimulus, the molecular mechanisms underlying the resulting developmental transition—here termed “cryometamorphosis”—remain poorly understood. Using an orthogonal experimental design, we categorized *A. ostoyae* as a “cold-pressed” species. Induction at 4 °C significantly accelerated primordium formation, reducing the time required for initiation from 25 to 7 days. Transcriptomic profiling across developmental and morphological stages revealed that primordium initiation was associated with the most extensive transcriptional reprograming across the reproductive trajectory. Weighted gene co-expression network analysis identified a core low-temperature-associated module, MEturquoise, and revealed a putative hierarchical regulatory network. Within this network, a group of fungus-specific Zn(II)_2_Cys_6_ transcription factors whose promoters contained predicted low-temperature response elements were tightly co-expressed with putative SET-domain histone methyltransferases and F-box proteins. To functionally validate this regulatory model, we established an optimized genetic transformation system for *A. ostoyae* and overexpressed the leading candidate gene, *AoZCy6_17*. Overexpression of *AoZCy6_17* at a constant temperature of 25 °C was associated with a pronounced morphological transition: no visible rhizomorph formation was observed in the OE strain under the conditions tested, together with increased production of dense aerial mycelia accompanied by abundant surface droplets. Biochemical assays showed that the overexpression strain exhibited enhanced basal antioxidant activity, with significantly higher catalase activity than the wild type (approximately 2,436 versus 2,001 U g^-1^ fresh weight; *P* < 0.001). This constitutive physiological reprograming was also associated with increased malondialdehyde accumulation under non-stress conditions (approximately 23.1 versus 9.6 nmol g^-1^ fresh weight), indicating a potential physiological cost. Notably, activation of *AoZCy6_17* alone recapitulated and, for some biochemical traits, exceeded the changes induced by exposure of the wild-type strain to 4 °C. Collectively, our findings provide functional evidence that *AoZCy6_17* is a key regulator linking low-temperature signaling to developmental and physiological reprograming in *A. ostoyae*, thereby establishing a mechanistic framework for investigating fungal cryometamorphosis.

## Introduction

The morphogenesis of fruiting bodies in mushroom-forming fungi is a highly regulated process accompanied by extensive stage-specific transcriptional changes and potential epigenetic regulation (Plaza et al. 2014; Song et al. 2018; Nagy et al. 2023). Although conserved regulatory gene families have been identified (Bayry et al. 2012; Todd et al. 2022; Yao et al. 2025), a critical gap remains: how are environmental cues integrated into these endogenous networks to trigger primordium initiation? For many basidiomycetes in temperate and boreal ecosystems, a seasonal decline in ambient temperature serves as an important, and in some species required, trigger for reproductive development, coordinating fruiting and spore dispersal with seasonal environmental conditions (Boddy et al. 2014; Andrew et al. 2018; Sakamoto 2018).

Traditionally, this transition has been viewed primarily through the lens of external environmental changes. However, recent biophysical advances have revealed a more complex thermal environment: mushroom-forming fungi can maintain temperatures below those of their surroundings (Cordero et al. 2023). Localized cooling has also been observed at the mycelial level and at presumptive fruiting sites before macroscopic development (Dressaire et al. 2016). We hypothesize that this trigger may involve a dynamic integration in which external thermal changes interact with localized cooling within the fungus to reach a critical signaling threshold. Because macroscopic morphogenesis is a prolonged process, a brief cold exposure alone may not be sufficient to complete this developmental transition. Rather than inducing only a transient stress response, low-temperature exposure may establish a sustained physiological state of temperature priming. Such a response could provide the temporal window required for stable transcriptional and chromatin-associated changes (Andrade-Linares et al. 2016). However, whether a discrete molecular switch exists to sense these thermal cues and initiate the large-scale transcriptional reprograming associated with cryometamorphosis remains an open question.

Given the complexity of this low-temperature induction system, existing terminology does not fully capture its developmental nature. In molecular studies, this transition is frequently described using the general term “cold stress” (e.g., Yan et al. 2016), which primarily refers to physiological responses to adverse conditions rather than an active commitment to reproductive development. Cultivation studies often use phenomenological terms such as “cold shock” or “cold stimulation” (Eastwood et al. 2013; Fu et al. 2016; Gong et al. 2022). Although useful for describing cultivation treatments, these terms remain largely descriptive and do not encompass the extensive molecular reprograming associated with the transition from vegetative growth to reproductive development.

To address this conceptual limitation, we propose the term “cryometamorphosis.” Functionally analogous, although not necessarily mechanistically identical, to plant vernalization (Penfield 2008; Andrés and Coupland 2012), cryometamorphosis describes morphogenetic reprograming triggered by exposure to a critical low-temperature regime. Its conceptual value lies in describing how external temperature cues interact with endogenous developmental programs and thermoregulatory processes to promote the formation of complex macroscopic multicellular structures from relatively simple vegetative networks (Knoll 2011; Nagy et al. 2018).

In natural habitats, this thermal requirement may act as an ecological safeguard against transient environmental fluctuations, thereby reducing phenological mismatches and unnecessary energetic investment (Boddy et al. 2014; Andrew et al. 2018). Because low-temperature-associated reproductive development occurs in many temperate and boreal basidiomycetes, elucidating its underlying molecular networks is of broad biological significance. However, these upstream regulatory pathways remain largely unexplored, particularly in ecologically and economically important forest pathogens such as *Armillaria ostoyae* (Li et al. 2023; Orban et al. 2024; Lin et al. 2024; Wang et al. 2025).

This study aimed to investigate the molecular mechanisms of cryometamorphosis in *A. ostoyae*. By integrating orthogonal cultivation experiments, transcriptomic profiling across developmental and morphological stages, weighted gene co-expression network analysis (WGCNA), promoter cis-regulatory element analysis, and functional validation through *Agrobacterium tumefaciens*-mediated transformation, we tested the following hypotheses:

Low-temperature induction acts as an initiating cue for *A. ostoyae* fruiting body development and is accompanied by stage-specific transcriptional reprograming and changes in candidate chromatin-associated regulators;

The transition from primordium initiation to fruiting body maturation involves coordinated transcriptional changes in thermally responsive and morphogenesis-related genes, including hydrophobins;

A central co-expression module involving Zn(II)_2_Cys_6_ transcription factors connects low-temperature signaling with the developmental program underlying cryometamorphosis, and the functional contribution of this module can be evaluated through targeted overexpression of a leading candidate regulator.

## Materials and methods

### Strains and cultivation conditions

**Figure 1.**
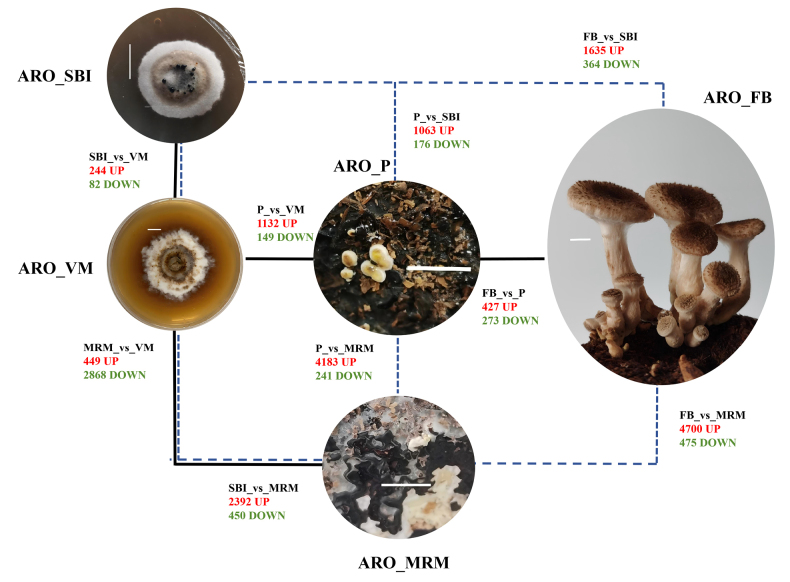
Transcriptomic changes across developmental and morphological states of *Armillaria ostoyae* JG19016. Solid lines indicate comparisons along the reproductive developmental sequence, whereas dashed lines indicate comparisons involving distinct, non-sequential morphological states. Red and green arrows indicate the numbers of significantly upregulated and downregulated genes, respectively (FDR < 0.05 and |log_2_FC| ≥ 2). ARO_VM, vegetative mycelium; ARO_P, primordium; ARO_FB, mature fruiting body; ARO_MRM, melanized rhizomorph; and ARO_SBI, monokaryotic mycelium. Three independent biological replicates were sequenced for each state. Scale bars: 1 cm.

The diploid *Armillaria ostoyae* strain JG19016, originally isolated from Heilongjiang Province, China, and its derived monokaryon (haploid) isolate JG19016/8 were used in this study. Both strains are maintained at the Engineering Research Center of the Chinese Ministry of Education for Edible and Medicinal Fungi, Jilin Agricultural University. Artificial bag cultivation for the induction of *A. ostoyae* fruiting bodies was performed on a solid substrate under previously described conditions (Chen et al. 2023). Samples representing distinct developmental and morphological stages were collected as shown in Fig. 1. These included vegetative mycelia grown on potato dextrose agar plates overlaid with cellophane (ARO_VM), primordia less than 1 cm in diameter (ARO_P), mature fruiting bodies with cap diameters of 7.5–8.5 cm (ARO_FB), and melanized rhizomorphs (ARO_MRM). Fruiting body stages were defined to conform to the general notation of mushroom developmental stages as closely as possible (Kües and Navarro-Gonzalez 2015).

The monokaryon isolate JG19016/8 (ARO_SBI) was cultured on Roth and Shaw medium containing 40 g L^-1^ malt extract, 20 g L^-1^ glucose, 5 g L^-1^ peptone, and 19 g L^-1^ agar. The plates were overlaid with cellophane and incubated at 25 °C for 3 weeks before sample collection. Three independent biological replicates were collected for each developmental or morphological stage. All samples were immediately frozen in liquid nitrogen and stored at −80 °C until RNA extraction.

### RNA sequencing and data processing

Total RNA extraction, library preparation, and paired-end sequencing on the Illumina HiSeq platform were performed by Sangon Biotech Co., Ltd. (Shanghai, China). Before library construction, RNA integrity and purity were assessed by gel electrophoresis and spectrophotometry. The quality of the raw sequencing reads was evaluated using FastQC. Adapter-contaminated and low-quality reads were subsequently removed during read preprocessing. The resulting clean reads were aligned to the *A. ostoyae* reference genome assembly GCA_900157425.1 using HISAT2 (Suppl. material 1: table SS1). Gene expression levels were quantified using StringTie v1.3.4 and expressed as transcripts per million reads (TPM) (Pertea et al. 2015). Raw gene-level count data were used for differential expression analysis. Functional annotations were assigned by searches against the NCBI non-redundant protein, Swiss-Prot, Gene Ontology (GO), and Pfam databases. Pearson correlation coefficients were calculated to assess relationships among biological replicates, and principal component analysis was performed to evaluate global variation in gene expression. Differentially expressed genes (DEGs) were identified using the DESeq2 package. To identify stage-associated transcriptional changes while limiting background variation, genes with a false discovery rate (FDR) below 0.05 and an absolute log_2_ fold change of at least 2 were considered differentially expressed. This threshold corresponded to a fold change greater than four and was selected with reference to previous transcriptomic analyses of *Armillaria ostoyae* species (Sipos et al. 2017).

GO and Kyoto Encyclopedia of Genes and Genomes (KEGG) enrichment analyses were performed for the identified DEGs using a hypergeometric test. Terms and pathways with significant overrepresentation were used to characterize the principal biological processes associated with each comparison. Heatmaps showing dynamic gene-expression patterns were generated using TBtools (Chen et al. 2023).

### Weighted gene co-expression network analysis

Gene co-expression networks were constructed using the WGCNA package in R (Langfelder and Horvath 2008). To improve network stability and reduce the contribution of genes with limited variation, the 5,000 genes with the highest median absolute deviation values were retained for network construction. Modules associated with low-temperature-induced fruiting body development were identified, and candidate hub genes were selected from the module showing the strongest association with this developmental response. To examine predicted functional associations among candidate genes, the protein sequences of the selected hub genes and additional candidate genes were searched against the Search Tool for the Retrieval of Interacting Genes/Proteins database (STRING) (Szklarczyk et al. 2020). The resulting protein–protein association network was imported into Cytoscape v3.10.1 for visualization (Zinovyev et al. 2008). Topologically important nodes were ranked using the CytoHubba plugin.

### AoZCy6 transcription factor family analysis

Members of the Zn(II)_2_Cys_6_ transcription factor family were identified by searching the *A. ostoyae* protein database against the hidden Markov model profile PF00172 using HMMER. Candidate proteins were subsequently examined using the NCBI Conserved Domain Database and Pfam to confirm the presence of the characteristic fungal Zn(II)_2_Cys_6_ DNA-binding domain.

A phylogenetic tree was reconstructed in MEGA v6.0 using the neighbor-joining method. Branch support was evaluated using 1,000 bootstrap replicates. To identify potential cis-regulatory elements, sequences extending 2,000 bp upstream of the predicted translation start sites were retrieved as putative promoter regions and analyzed using PlantCARE. Particular attention was paid to predicted low-temperature-responsive elements.

Potential downstream target genes were further inferred by searching the promoter regions of cold-responsive genes in the MEturquoise module for the conserved GAL4-type binding motif MA0299.1 obtained from the JASPAR database.

### RNA extraction, cDNA synthesis, and quantitative real-time PCR

Total RNA was extracted using the UNIQ-10 Column TRIzol Total RNA Extraction Kit (Sangon Biotech, Shanghai, China), and RNA concentration and purity were measured using a NanoDrop spectrophotometer. Complementary DNA was synthesized from total RNA and used as the template for quantitative real-time PCR (qPCR).

Because samples collected across fruiting-body developmental stages and during the low-temperature time-course experiment represented distinct physiological states and temperature conditions, the expression stability of candidate reference genes could not be ensured. Therefore, an equal-RNA-input normalization strategy was used for these experiments. Exactly 1,000 ng of total RNA from each biological replicate was reverse-transcribed under identical reaction conditions, and equivalent amounts of cDNA were used for qPCR. Relative transcript abundance was calculated as 2^−(Ctsample−Ctcalibrator)^, using vegetative mycelium as the calibrator for developmental-stage comparisons and day 0 as the calibrator for the low-temperature time-course experiment. For comparisons between the overexpression treatment and the corresponding control, relative transcript abundance was calculated using the 2^−ΔΔCt^ method, with GAPDH as the internal reference gene. Technical replicate measurements were required to agree within 0.5 Ct cycles; discordant measurements were excluded from the final calculation.

### Statistical analysis of qPCR data

Statistical analyses were performed using Ct-based values rather than normalized fold-change values. For developmental-stage and low-temperature time-course experiments, matched Ct differences relative to the corresponding calibrator were evaluated against zero using two-tailed one-sample Student’s t-tests. For the overexpression experiment, ΔCt values normalized to GAPDH were compared between the WT and OE groups using a two-tailed Student’s t-test. All statistical analyses and graphical visualizations were performed in R using the ggplot2 and ggsignif packages. Statistical significance was indicated as follows: *P < 0.05, **P < 0.01, and ***P < 0.001.

### Vector construction and fungal transformation

An overexpression vector for *AoZCy6_17* was constructed by amplifying the native GPD promoter from genomic DNA and the full-length coding sequence of *AoZCy6_17* from the cDNA derived from *A. ostoyae* JG19016. The purified amplicons were assembled into the binary vector pCAMBIA1302 using a Seamless Cloning Kit (Biomed, Beijing, China). The recombinant plasmid, pCAMBIA1302-*AoZCy6_17*, was propagated in *Escherichia coli* TOP10 competent cells and verified by Sanger sequencing. The verified plasmid was subsequently introduced into *Agrobacterium tumefaciens* strain EHA105 using the freeze-thaw method.

Fungal transformation was performed using *A. tumefaciens*-mediated transformation. Briefly, *A. tumefaciens* carrying the overexpression construct was cultured to OD_600_ approximately 1.0 and resuspended in liquid medium supplemented with 150 µmol L^-1^ acetosyringone. Fresh mycelial plugs excised from actively growing colony margins were immersed in the bacterial suspension and incubated at 25 °C with shaking at 120 rpm for 10 h. After co-cultivation, the mycelial plugs were washed with sterile deionized water and transferred to selective medium containing 50 μg mL^-1^ hygromycin B. Putative transformants were isolated and verified by genomic PCR and RT-qPCR. The primers used for vector construction and transformant verification are listed in Suppl. material 1: table S22.

### Phenotypic and biochemical assays of transformants

The macroscopic colony morphologies of the wild-type and *AoZCy6_17*-overexpressing strain were photographed after cultivation on solid MYG medium at 25 °C. Fresh mycelial samples maintained at 25 °C or subjected to low-temperature induction at 4 °C were collected for biochemical analyses. Catalase activity and malondialdehyde content were measured using commercial assay kits according to the manufacturer’s instructions. The catalase assay was performed using kit BC0205, and malondialdehyde content was measured using kit BC6415 (Solarbio Science & Technology Co., Ltd., Beijing, China).

## Results

### Low-temperature induction is a critical determinant of primordium initiation and developmental rate in Armillaria ostoyae

A three-factor, three-level orthogonal experiment was conducted to evaluate the effects of temperature regime, light duration, and exogenous supplementation on fruiting body development in mature *A. ostoyae* mycelia (Table 1). No primordia were observed in the groups maintained continuously at 25 °C (Groups 1–3). By contrast, primordia were observed in each treatment group cultured at 15 °C, either with or without prior exposure to 4 °C (Groups 4–9). These results indicate that, under the conditions tested, low-temperature treatment was required for primordium initiation in *A. ostoyae*.

**Table 1.** Optimization of fruiting body production in *Armillaria ostoyae* using a three-factor, three-level orthogonal experimental design.

**Group**	**A: Temperature regime (°C)**	**B: Supplementation treatment**	**C: Light (h day^-1^)**	**Total fresh weight (g)**
Group 1	25	0 mL	0	0
Group 2	25	50 mL sterile water	6	0
Group 3	25	50 mL nutrient solution	12	0
Group 4	15	0 mL	12	13.37 ± 6.54 c
Group 5	15	50 mL sterile water	6	26.49 ± 2.89 bc
Group 6	15	50 mL nutrient solution	0	24.79 ± 6.06 bc
Group 7	4 °C → 15 °C	0 mL	6	46.27 ± 5.33 a
Group 8	4 °C → 15 °C	50 mL sterile water	0	20.38 ± 5.72 bc
Group 9	4 °C → 15 °C	50 mL nutrient solution	12	28.52 ± 5.14 b
K1	0	59.64	45.17	
K2	64.65	46.87	72.76	
K3	95.17	53.31	41.89	
Best level	3	1	2	
R	95.17	12.77	30.87	

**Note**: A value of 0 indicates that no fruiting bodies were produced. Different lowercase letters indicate significant differences in total fresh weight among treatment groups according to Tukey’s HSD test (*P* < 0.05). Values are presented as the mean ± standard deviation of three independent replicates. K_1_–K_3_ represent the sums of the response values at the three levels of each factor, and R represents the difference between the maximum and minimum K values.

The temperature regime also markedly affected the time required for primordium formation (Table 2). Mycelia maintained continuously at 15 °C (Groups 4–6) formed primordia after 25 days. In contrast, mycelia pretreated at 4 °C for 3 days before transfer to 15 °C (Groups 7–9) formed primordia after only 7 days. Thus, the 4 °C pretreatment shortened the time to primordium initiation by 18 days. Range analysis based on total fruiting body fresh weight identified temperature as the factor with the greatest effect (R = 95.17), followed by light duration (R = 30.87) and supplementation treatment (R = 12.77) (Table 1).

**Table 2.** Effects of different cultivation treatments on primordium development and fruiting body traits in *Armillaria ostoyae*.

**Treatment group**	**Time to primordium formation (d)**	**Primordium-to-fruiting conversion rate (%)**	**Pileus diameter (cm)**	**Biological efficiency (%)**
Group 1	–	–	–	–
Group 2	–	–	–	–
Group 3	–	–	–	–
Group 4	25	100	4.40 ± 0.36 b	1.37 ± 0.67 d
Group 5	25	71.43	4.37 ± 0.31 b	2.71 ± 0.30 b
Group 6	25	28.57	4.23 ± 0.31 b	2.54 ± 0.62 bc
Group 7	7	100	3.80 ± 0.20 b	4.74 ± 0.55 a
Group 8	7	85.71	5.30 ± 0.36 a	2.09 ± 0.59 c
Group 9	7	57.14	4.37 ± 0.21 b	2.92 ± 0.53 b

**Note**: An em dash indicates that no primordia or fruiting bodies were produced. The primordium-to-fruiting conversion rate was calculated as the number of cultivation units that produced fruiting bodies divided by the number of cultivation units in which primordia had formed, multiplied by 100. Within each continuous-variable column, values followed by different lowercase letters differ significantly according to Tukey’s HSD test (P < 0.05). Pileus diameter and biological efficiency are presented as the mean ± standard deviation of three independent replicates.

The different treatment combinations also affected the subsequent progression of primordia into mature fruiting bodies, as well as total fresh weight, biological efficiency, and pileus diameter. Group 7, which combined a 4 °C pretreatment with 6 h of light per day and no exogenous supplementation, exhibited a 100% primordium-to-fruiting conversion rate and produced the highest total fresh weight (46.27 ± 5.33 g). The total fresh weight in Group 7 was significantly higher than that in all other groups (*P* < 0.05) and was approximately 3.5-fold higher than that in Group 4 (13.37 ± 6.54 g). Group 7 also exhibited the highest biological efficiency, reaching 4.74 ± 0.55% (Table 2).

Despite producing the highest total fresh weight and biological efficiency, Group 7 had the smallest mean pileus diameter among the fruiting-body-forming treatments (3.80 ± 0.20 cm). In contrast, Group 8 produced the largest mean pileus diameter (5.30 ± 0.36 cm), which was significantly greater than those observed in the other fruiting-body-forming groups (*P* < 0.05), but had substantially lower total fresh weight and biological efficiency than Group 7. These results indicate that low-temperature exposure was the principal determinant of primordium initiation and developmental timing, whereas the complete cultivation regime influenced subsequent fruiting development, yield, and fruiting body morphology. In particular, the Group 7 treatment favored the successful progression of primordia into mature fruiting bodies and increased overall fruiting body production rather than promoting the expansion of individual pilei. Replicate-level measurements of pileus diameter and total fresh weight are provided in Suppl. material 1: table SS2.

### Expression profiles of primordia and fruiting bodies

Although low temperature serves as an important morphogenetic trigger for sexual reproduction, the molecular networks underlying low-temperature-induced fruiting body development remain poorly understood. To characterize the principal transcriptional changes associated with this developmental process, differentially expressed genes (DEGs) were identified using thresholds of FDR < 0.05 and |log_2_FC| ≥ 2 (Fig. 1). Pairwise transcriptomic comparisons among the developmental stages and vegetative or morphological states showed that the transition from vegetative mycelium (ARO_VM) to primordium formation (ARO_P) involved the most extensive transcriptional changes (Suppl. material 1: tables S3–S6). Genes upregulated during primordium formation were significantly enriched in Gene Ontology (GO) terms associated with responses to stimuli, protein kinase activity, mitotic and meiotic processes, carbohydrate and lipid metabolism, and pectin catabolism (Suppl. material 1: table S7). By contrast, the transition from primordia to mature fruiting bodies (ARO_FB) involved fewer transcriptional changes (Fig. 1; Suppl. material 1: table S4). Nevertheless, genes upregulated in ARO_FB relative to ARO_P were enriched in GO terms associated with responses to stimuli, protein kinase activity, endoplasmic reticulum membrane components, regulation of meiosis and sporulation, and G-protein-coupled receptor activity (Suppl. material 1: table S8).

Consistent with the relatively small number of differentially expressed genes, global expression profile analysis showed that the transcriptomes of ARO_P and ARO_FB clustered closely together (Suppl. material 2: fig. S1). Similar expression patterns were also observed across several putative stimulus-responsive gene families and individual genes, including fatty acid desaturases, phospholipases, and components of calcium signaling pathways (Suppl. material 2: fig. S2).

A total of 39 genes were significantly upregulated in both ARO_P and ARO_FB relative to ARO_VM (FDR < 0.05 and log_2_FC ≥ 2 in both comparisons; Suppl. material 1: table S9). This shared set of development-associated genes included genes encoding SET-domain proteins, fungal chitosanases, C3HC4-type zinc finger proteins, cytochrome P450 enzymes, and members of the 2-oxoglutarate/Fe(II)-dependent oxygenase superfamily.

Several protein kinase genes (ARMOST_06811, ARMOST_06804, ARMOST_05978, and ARMOST_01461) and candidate chromatin-associated regulators were also upregulated. The latter included genes encoding putative SET-domain histone methyltransferases (ARMOST_11846, ARMOST_11860, ARMOST_11913, and ARMOST_11914) and lysine methyltransferases (ARMOST_06760 and ARMOST_06758), suggesting that chromatin-associated regulation may contribute to the developmental transition. Genes encoding RING-type zinc finger proteins (ARMOST_00102, ARMOST_19694, and ARMOST_16435) and an F-box protein (ARMOST_20619) were also upregulated, consistent with a potential role for ubiquitin-mediated protein turnover during fruiting body development.

In addition to this shared expression subset, 393 genes exhibited a primordium-enriched expression pattern. These genes were significantly upregulated in ARO_P relative to ARO_VM and subsequently downregulated in ARO_FB relative to ARO_P (Suppl. material 1: table S10). Among the most strongly induced genes in this group were hydrophobin genes, including one with a log_2_FC of 18.21, RicinB_lectin domain-containing genes, and genes encoding members of several transcription factor families, including Zn(II)_2_Cys_6_ and zf-MYND transcription factors. The primordium stage contained the largest number of upregulated genes encoding predicted transcription factors among the developmental and morphological states examined (Suppl. material 2: fig. S3A). The expression profiles of these primordium-enriched candidates are presented in Suppl. material 1: table S11 and are consistent with extensive transcriptional reprograming during low-temperature-induced primordium formation.

Hydrophobin genes also exhibited a pronounced stage-associated expression pattern, with most reaching their highest expression levels during the primordium stage. Hydrophobins are important cell-surface proteins associated with aerial hypha formation and primordium assembly (Xu et al. 2021). Their concurrent enrichment with genes encoding predicted transcription factors was consistent with coordinated structural and transcriptional changes during primordium formation. A distinct but partially overlapping set of predicted transcription factors and hydrophobin genes was also differentially expressed in the monokaryotic mycelial state (ARO_SBI), which was similarly characterized by abundant aerial hyphae.

### Expression profiles of melanized rhizomorphs

The rhizomorph, a multicellular structure unique to *Armillaria* spp., remains a subject of debate regarding its precise biological function and morphogenesis (Sipos et al. 2017; Porter et al. 2022). In this study, we observed that fruiting bodies produced under artificial cultivation conditions preferentially developed from melanized rhizomorphs. To elucidate their molecular characteristics, we performed transcriptomic analysis of mature melanized rhizomorphs (ARO_MRM) collected from the surface of the cultivation substrate.

Transcriptomic comparisons between melanized rhizomorphs and the reproductive tissues represented by primordia and fruiting bodies revealed a predominance of genes expressed at lower levels in the rhizomorphs, with only a restricted subset of genes exhibiting significant upregulation (FDR < 0.05 and |log_2_FC| ≥ 2; Fig. 1). Gene Ontology enrichment analysis indicated that these upregulated genes were significantly enriched in GO terms related to responses to stimuli, regulation of carbohydrate and lipid metabolism, and cell-wall growth. These enrichment patterns were consistent with roles in defense and metabolic regulation during responses to external environmental stress (Suppl. material 1: table S12).

Global expression profiling revealed that the transcriptome of melanized rhizomorphs was distinct from the main developmental sequence, which progresses from vegetative mycelium to primordia and mature fruiting bodies (Suppl. material 2: fig. S1). Key morphogenesis-related genes, including those involved in cell-wall biosynthesis, GMC oxidoreductases, and pore-forming toxins, were distinctly regulated in rhizomorphs. Nonetheless, selected cell-wall protein genes, such as specific expansins and hydrophobins, maintained notable expression in this structure, with some clustering closely with the primordium-enriched cohort (Suppl. material 2: fig. S3B; Suppl. material 1: table S13). This expression overlap suggests that melanized rhizomorphs retain a subset of molecular functions potentially relevant to fruiting body development, particularly those associated with hyphal adhesion, intercellular communication, and defense.

Furthermore, we identified nine rhizomorph-upregulated genes annotated as being associated with responses to external stimuli (Suppl. material 1: table S13). This repertoire included the transcription elongation factor SPT6, ribosomal protein S6 kinase, silencing information regulator 5 and related SIR2-family proteins, an SNF2-family DNA-dependent ATPase, proteins involved in rRNA processing, serine/threonine protein kinases, and sensory transduction histidine kinases. Concurrently, we detected seven significantly upregulated genes encoding predicted transcription factors in this structure. Interestingly, five of these genes were also upregulated in primordia. For example, the putative basic leucine zipper transcription factor gene ARMOST_01134 and the predicted Zn(II)_2_Cys_6_ transcription factor gene ARMOST_00729 exhibited high expression in rhizomorphs and primordia and remained highly expressed in mature fruiting bodies. Their expression across these states suggests potential roles in regulatory processes shared among rhizomorph development, environmental stress responses, and reproductive morphogenesis.

### Expression profiles of the monokaryotic isolate

Germination of basidiospores to form monokaryotic mycelia marks the beginning of the life cycle in most mushroom-forming fungi. Subsequently, two compatible monokaryons fuse to form a fertile dikaryotic mycelium. In *Armillaria*, monokaryotic isolates can produce abundant aerial mycelia that are morphologically similar to primordia and fruiting bodies. Differential expression analysis of the monokaryotic isolate (ARO_SBI) revealed relatively few transcriptional differences compared with vegetative mycelia. However, comparisons with the reproductive tissues represented by primordia and mature fruiting bodies revealed a predominance of genes expressed at lower levels in ARO_SBI, with only a limited subset exhibiting significant upregulation (FDR < 0.05 and |log_2_FC| ≥ 2; Fig. 1). By contrast, comparison with melanized rhizomorphs revealed a relatively larger number of upregulated genes and fewer downregulated genes in ARO_SBI (Fig. 1; Suppl. material 1: table S14).

Gene Ontology (GO) enrichment analysis of genes upregulated in ARO_SBI in the four pairwise comparisons revealed significant enrichment of GO terms associated with stress responses and metabolism, including oxidation–reduction processes, peroxidase activity, gluconeogenesis, and phosphorus metabolism (Suppl. material 1: table S15). This functional profile was consistent with distinct stress-response and metabolic features in monokaryotic mycelia compared with fruiting bodies and melanized rhizomorphs. Global expression profiling demonstrated that the overall transcriptional profile of ARO_SBI clustered closely with that of vegetative mycelia and was distinct from the expression profiles associated with complex multicellular structures (Suppl. material 2: fig. S1). The inability of an isolated monokaryon to complete fruiting body differentiation without a compatible mating partner was consistent with its putative morphogenesis-related gene profiles, which largely resembled those of vegetative mycelia. However, ARO_SBI showed upregulation of a distinct set of genes encoding predicted transcription factors, hydrophobins, and oxidoreductases (Suppl. material 2: figs S3, S4). This localized upregulation may be associated with the abundant aerial hyphae, which are directly exposed to external environmental fluctuations. Overall, the transcriptional landscape of ARO_SBI represented a distinct developmental and physiological state characterized by a vegetative-mycelium-like global profile together with specialized expression of genes potentially associated with environmental responses.

### Analysis of core regulators in fruiting body development under low-temperature induction

**Figure 2.**
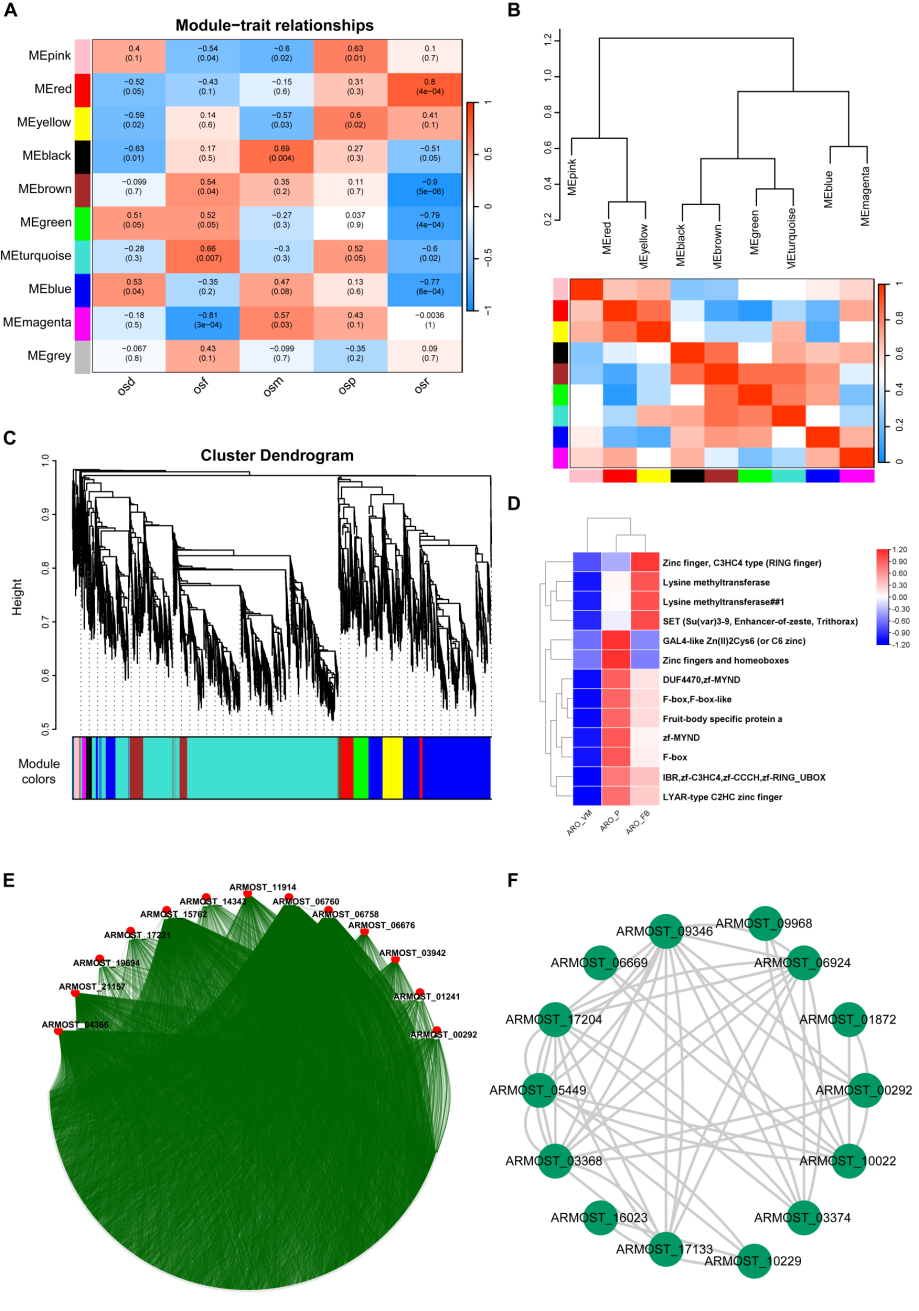
Weighted gene co-expression network analysis and identification of hub genes. **A** Associations between WGCNA modules and low-temperature-induced fruiting body development. Cell colors indicate correlation coefficients, with blue representing negative correlations and red representing positive correlations. *P* values are shown in parentheses **B** Eigengene adjacency clustering and heatmap of module eigengene adjacency **C** Gene clustering dendrogram based on topological overlap, with the assigned module colors shown below. Each leaf represents a gene, and branch height indicates dissimilarity between genes **D** Heatmap showing the expression patterns of hub genes in the MEturquoise module **E** Identification of hub genes within the MEturquoise module using the CytoHubba application in Cytoscape **F** Predicted protein–protein association network of candidate genes generated using the STRING database.

To systematically characterize the co-expression networks associated with primordium induction and fruiting body development under low-temperature conditions, weighted gene co-expression network analysis (WGCNA) was performed using the 5,000 genes with the highest median absolute deviation (MAD) across all transcriptomic samples. Hierarchical clustering identified 10 distinct co-expression modules (Fig. 2C), and module eigengene adjacency analysis revealed the structural relationships among these modules (Fig. 2B). Notably, the MEturquoise module exhibited the strongest positive correlation with the fruiting body stage (*r* = 0.665, *P* = 0.007) and maintained a moderate positive correlation with the primordium stage (*r* = 0.517, *P* = 0.05) (Fig. 2A).

To identify candidate developmental regulators, hub genes within the MEturquoise module were extracted, and their expression patterns were visualized (Fig. 2D). Analysis of the co-expression network topology using Cytoscape identified 13 primary hub genes (Fig. 2E). Further network-based expansion identified an additional 55 closely associated candidate genes. Together, this set of 68 genes (Suppl. material 1: table S16) included genes annotated as being involved in transcriptional regulation, including Zn(II)_2_Cys_6_- and C2HC/C2H2-type transcription factors; chromatin modification, including SET-domain histone lysine methyltransferases; and ubiquitin-mediated protein degradation, including F-box components of E3 ubiquitin ligases.

To investigate the potential functional associations among these 68 genes, their predicted protein associations were examined using the STRING database. This analysis yielded a connected subnetwork consisting of 13 nodes and 20 edges at a combined interaction score > 0.4. The network was enriched in functions related to RNA polymerase III transcription and included predicted associations among several candidate morphogenesis-related factors, including Zn(II)_2_Cys_6_ and C2H2 transcription factors, a SET-domain methyltransferase, and the RNA polymerase subunit Rpb5 (Fig. 2F). Together, the WGCNA correlations and STRING-predicted associations support coordinated expression and potential functional relationships among these chromatin-associated and transcriptional regulators, thereby contributing to the cryometamorphosis expression patterns associated with fruiting body development in *Armillaria ostoyae*.

### Structural characterization of Zn(II)_2_Cys_6_ transcription factors

**Figure 3.**
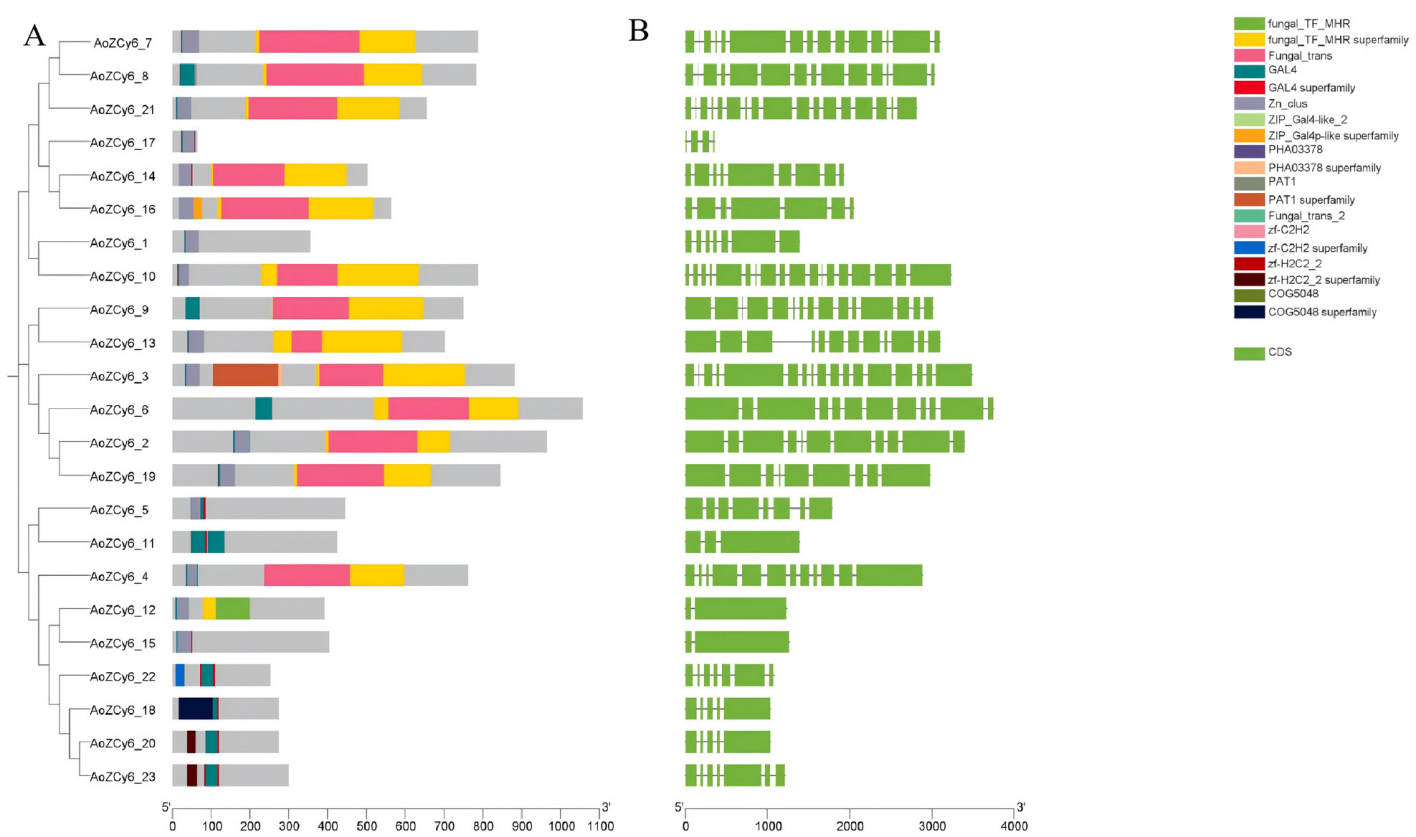
Phylogenetic and structural characterization of Zn(II)_2_Cys_6_ transcription factors in *Armillaria ostoyae*. **A** Phylogenetic tree constructed using the neighbor-joining method in MEGA 6.0 based on protein sequences retrieved from the NCBI database and aligned using ClustalW. The amino acid sequences of the GAL4-like DNA-binding domains are shown, with conserved residues highlighted in different colors **B** Schematic representation of the conserved domains and gene structures of C6 homologs in *A. ostoyae*, with corresponding features indicated by colored boxes.

Zn(II)_2_Cys_6_ (C6) transcription factors represent a fungus-specific subclass of zinc finger proteins characterized by a highly conserved C6 domain (Cys-X2-Cys-X6-Cys-X5-12-Cys-X2-Cys-X6-9-Cys), which coordinates zinc ions to form a functional DNA-binding domain (Chang and Ehrlich 2013). Originally identified in *Saccharomyces cerevisiae* GAL4 protein, this family classically functions in activating galactose metabolism-related genes (Kraulis et al. 1992). In *Armillaria ostoyae*, we identified 23 developmentally regulated Zn(II)_2_Cys_6_ transcription factors (designated AoZCy6_1 to 23), all encompassing the complete C6 conserved motif (Fig. 3, Suppl. material 1: table S17). Phylogenetic analysis classified these factors into five distinct evolutionary clades, each possessing the characteristic N-terminal GAL4-like DNA-binding domain. Gene structure analysis revealed that these AoZCy6 genes consist of 3 to 18 exons, with 12 specific genes notably harboring low-temperature response elements (LTREs) within their promoter regions (Suppl. material 1: table S18).

To delineate the regulatory network of these AoZCy6 factors, potential downstream targets were predicted by screening stress-related genes within the low-temperature-associated MEturquoise module. By analyzing the promoter regions of these module genes for the GAL4 binding motif (MA0299.1) using the JASPAR database, we identified a robust suite of candidate targets (Suppl. material 1: table S19). These targets span essential functional categories, including carbohydrate-active enzymes (e.g., GH47 and GT8 families, starch-binding domain proteins), oxidative stress scavengers (e.g., superoxide dismutase SOD and catalase CAT), mitochondrial functional regulators (e.g., eukaryotic mitochondrial regulatory proteins, DNA-dependent ATPases, and 5'–3' DNA helicases), circadian rhythm-associated factors (e.g., XAP5), and molecular chaperones (e.g., HSP90, HSP12, and HSP20). Collectively, these predicted target genes form an integrated transcriptional network, suggesting that AoZCy6 transcription factors orchestrate the coordinated control of stress responses, energy metabolism, and protein homeostasis critically required for cryometamorphosis.

To assess the consistency of the transcriptomic data, the relative expression profiles of ten representative ARMOST candidate genes were quantified by qRT-PCR across ARO_VM, ARO_P, and ARO_FB. Several candidates exhibited stage-associated expression changes that broadly agreed with the corresponding RNA-seq trends, whereas differences in expression magnitude were observed for some genes (Suppl. material 2: fig. S5; Suppl. material 1: table S21).

### Temporal transcriptional activation of cryometamorphosis candidate genes

To characterize the temporal expression responses associated with cryometamorphosis, we monitored the relative transcript levels of the core candidate transcription factor *AoZCy6_17* and three co-expressed candidate targets, ARMOST_01387, ARMOST_12835, and ARMOST_18251, using qPCR. Temporal profiling was performed using fully colonized mycelial cultivation bags subjected to low-temperature induction at 4 °C for 7 days.

**Figure 4.**
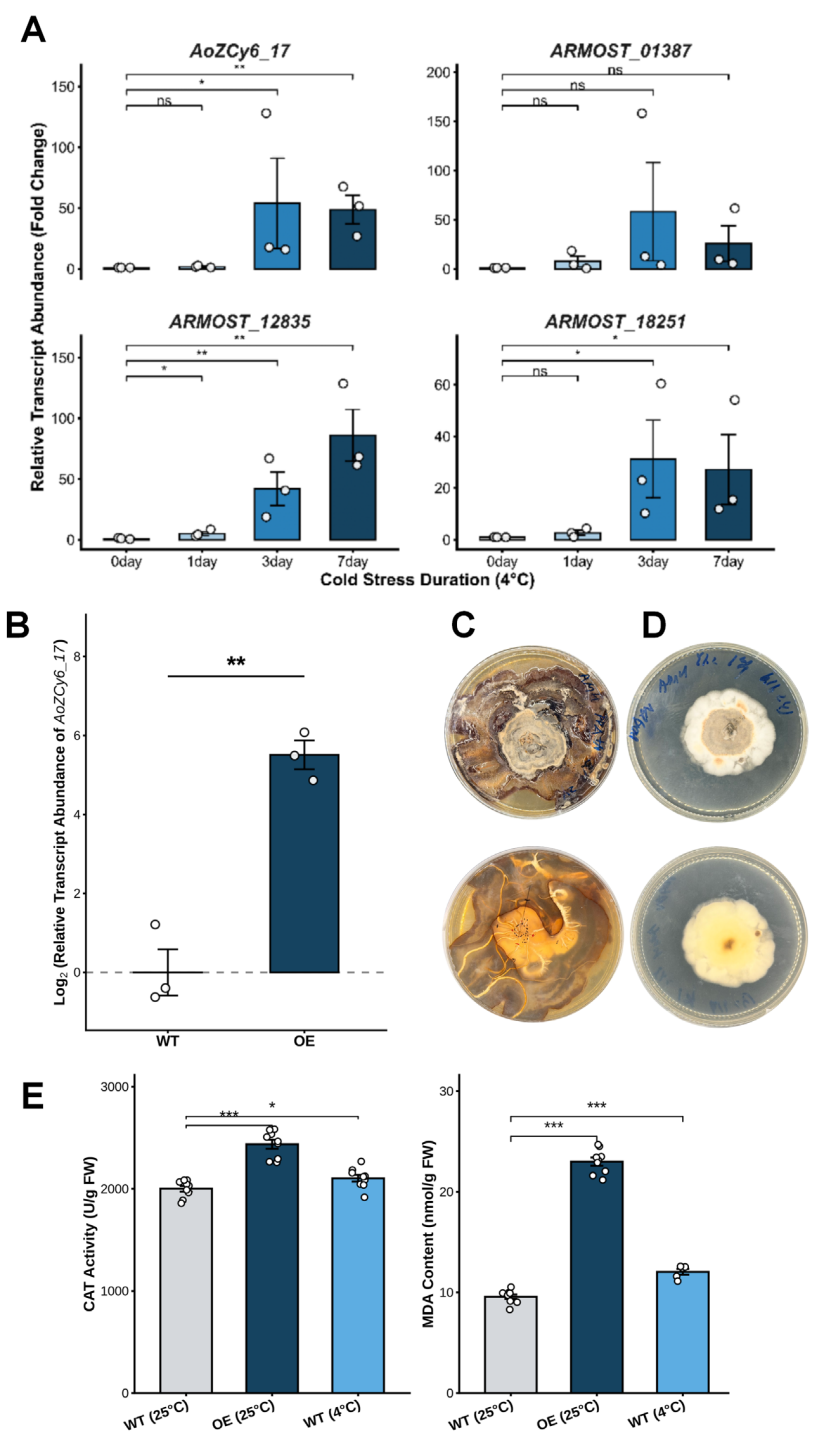
Temporal expression, overexpression validation, and morphophysiological phenotypes associated with the core candidate regulator *AoZCy6_17*. **A** Temporal expression profiles of *AoZCy6_17* and three co-expressed candidate genes (ARMOST_01387, ARMOST_12835, and ARMOST_18251) during a 7-day low-temperature induction at 4 °C. Relative transcript abundance was normalized to the day-0 baseline **B** RT-qPCR confirmation of elevated *AoZCy6_17* expression in the OE strain. The y-axis shows log_2_-transformed relative transcript abundance **C, D** Macroscopic colony morphologies of the WT (**C**) and OE (**D**) line cultivated at 25 °C. Compared with the characteristic rhizomorph networks produced by the WT strain, no visible rhizomorph formation was observed in the OE strain under the conditions tested; instead, the OE strain exhibited increased production of dense aerial mycelia and visible surface condensation **E** Catalase activity and malondialdehyde content in the WT strain at 25 °C, the OE strain at 25 °C, and the WT strain following low-temperature treatment. Statistical significance is indicated as follows: *P < 0.05, **P < 0.01, and ***P < 0.001; ns, not significant.

As shown in Fig. 4A and detailed in Suppl. material 1: table S21, most candidate genes showed no significant changes in transcript abundance during the first 24 h of low-temperature exposure. However, prolonged low-temperature induction was accompanied by marked increases in expression. Specifically, *AoZCy6_17* showed a 53.9-fold increase on day 3 (P = 0.0356) and remained elevated by 48.7-fold on day 7 (P = 0.0051). This cold-responsive expression pattern is consistent with the potential involvement of *AoZCy6_17* in low-temperature-associated developmental regulation and with the presence of predicted low-temperature-responsive elements (LTREs) in its promoter region, although the functional activity of these elements remains to be experimentally verified.

In parallel with the increased expression of *AoZCy6_17*, several co-expressed candidate genes associated with structural morphogenesis and antioxidant functions also showed increased transcript abundance during prolonged low-temperature induction. ARMOST_18251, annotated as encoding superoxide dismutase, showed increased transcript abundance on days 3 and 7. ARMOST_01387 also exhibited an overall trend of increased expression, although substantial variation was observed among biological replicates. ARMOST_12835 showed an apparent increase during prolonged low-temperature exposure. These results indicate variation in the magnitude and reproducibility of the transcriptional responses among the examined candidate genes.

Taken together, these temporal expression profiles provide experimental support for the expression relationships suggested by the WGCNA analysis. Although the data do not demonstrate direct regulatory interactions, they show that *AoZCy6_17* and several co-expressed candidate genes respond during prolonged low-temperature induction, consistent with a coordinated transcriptional transition associated with early cryometamorphosis in *Armillaria ostoyae*.

### Functional characterization of the core transcription factor *AoZCy6_17*

#### Generation and validation of an *AoZCy6_17* overexpression strain

To assess the functional contribution of the core candidate transcription factor *AoZCy6_17* (Suppl. material 2: fig. S6), we generated an *AoZCy6_17*-overexpressing (OE) strain. The presence of the overexpression construct was confirmed by genomic PCR, and RT-qPCR analysis using GAPDH as the internal reference gene showed increased *AoZCy6_17* transcript abundance in the OE strain relative to the wild-type (WT) strain (Fig. 4B; Suppl. material 1: table S23). These results confirmed transcriptional overexpression and provided the basis for subsequent phenotypic and biochemical analyses.

#### Morphological and biochemical phenotypes of the OE strain

Overexpression of *AoZCy6_17* was accompanied by pronounced changes in colony morphology (Fig. 4C, D). Whereas the WT strain developed characteristic rhizomorph networks, no visible rhizomorph formation was observed in the OE strain across the examined culture plates. Instead, the OE strain exhibited increased production of dense aerial mycelia. These observations indicate that elevated *AoZCy6_17* expression influences colony architecture and rhizomorph development under the conditions tested.

Biochemical analyses further revealed significant changes in oxidative-status indicators in the OE strain. At 25 °C, the OE strain exhibited significantly higher basal catalase (CAT) activity, with a mean of approximately 2,436 U/g FW, than the WT strain, which had a mean activity of approximately 2,001 U/g FW (*P* < 0.001; Fig. 4E left). Malondialdehyde (MDA) content, an indicator of lipid peroxidation, was also significantly higher in the OE strain, at approximately 23.1 nmol/g FW, than in the WT strain, at approximately 9.6 nmol/g FW (*P* < 0.001; Fig. 4E right). In the WT strain, low-temperature treatment at 4 °C also increased CAT activity to approximately 2,104 U/g FW and MDA content to approximately 12.0 nmol/g FW relative to the corresponding WT values at 25 °C (*P* < 0.05). Notably, the OE strain showed elevated CAT activity and MDA accumulation at 25 °C in the absence of low-temperature induction (raw data and statistical parameters are provided in Suppl. material 1: table S24). These results indicate that *AoZCy6_17* overexpression alters basal CAT activity and MDA accumulation and may influence the oxidative status of the mycelia before low-temperature induction.

## Discussion

The morphological transition from vegetative mycelia to fruiting bodies in basidiomycetes represents one of the most complex and energy-intensive processes in the fungal life cycle. This transformation entails not only profound cellular and morphological changes but also genome-wide transcriptional reprograming (Nagy et al. 2023). In the cultivation of edible and medicinal fungi, temperature is recognized as a pivotal environmental factor governing this developmental transition. Focusing on *Armillaria ostoyae*, a species of considerable ecological and economic importance, this study integrated cultivation-response data obtained from an orthogonal experiment with high-throughput RNA sequencing (RNA-seq) and weighted gene co-expression network analysis (WGCNA) to establish a molecular framework for low-temperature-associated fruiting body development. We further evaluated this framework by examining the cold-responsive expression dynamics of candidate genes through temporal transcriptional profiling and by assessing the phenotypic and biochemical changes associated with *AoZCy6_17* overexpression.

### Armillaria ostoyae as a cold-pressed type species

Characterizing physiological responses to environmental signals provides a foundation for elucidating the molecular mechanisms underlying fungal development. The orthogonal experimental results (Tables 1, 2) indicated that temperature was a major determinant of *A. ostoyae* fruiting body development under the conditions tested. Analysis of the responses across the temperature treatments revealed distinct developmental requirements. Although *A. ostoyae* exhibited vigorous vegetative growth at 25 °C, no primordia were observed under these conditions. By contrast, primordia appeared after 25 days at 15 °C, whereas a 3-day low-temperature pretreatment at 4 °C, followed by cultivation at 15 °C, reduced the time to primordium initiation to 7 days. Following the thermal classification system for edible fungi proposed by Huang et al. (2023), we categorize *A. ostoyae* as a “cold-pressed type” species, defined by a fruiting induction temperature of ≤10 °C, distinguishing it from “low-temperature type” species, such as *Agaricus bisporus*, which require only a moderate temperature decrease for fruiting induction.

This pronounced dependence on low-temperature induction may have ecological and evolutionary significance. As a long-lived forest pathogen, *A. ostoyae* relies on vegetative structures, including rhizomorphs and mycelia, for prolonged subterranean survival (Baumgartner et al. 2011). The requirement for sustained low-temperature exposure may function as a seasonal constraint that reduces premature initiation of energy-intensive reproductive development during transient cooling events in late summer or early autumn. This constraint may be relieved when sustained low temperatures resembling late-autumn conditions occur together with auxiliary environmental cues, such as light. Such a regulatory strategy may help align fruiting body emergence with environmental conditions favorable for reproductive development and spore dispersal.

### Stage-specific expression patterns: Transcriptional reprograming during primordium initiation

RNA-seq analysis revealed that the transition from vegetative mycelia (ARO_VM) to primordia (ARO_P) produced the largest number of differentially expressed genes (DEGs). By contrast, transcriptional changes during the subsequent transition from primordia to mature fruiting bodies (ARO_FB) were comparatively limited. This pattern suggests that the major transcriptional reprograming associated with reproductive development occurs during primordium initiation, whereas subsequent fruiting body development may involve a more restricted set of transcriptional changes. Once the developmental program leading to primordium formation has been initiated, later development may increasingly depend on coordinated tissue growth, cell expansion, and structural differentiation rather than on further genome-wide transcriptional reprograming (Nagy et al. 2023).

Gene Ontology (GO) enrichment analysis showed that genes upregulated during the primordium stage were enriched in terms including “response to stimulus,” “protein kinase activity,” and “cell wall organization or biogenesis.” These results are consistent with primordium formation involving coordinated environmental sensing, signal transduction, and cellular remodeling. The elevated expression of kinase-related genes, including putative components associated with MAPK signaling, suggests that phosphorylation-mediated signaling may contribute to the activation of downstream transcriptional responses. In parallel, the transition from filamentous vegetative hyphae to organized multicellular primordia requires substantial modification of polarized growth and cell-wall architecture. This developmental requirement is consistent with the enrichment of cell-wall-related genes, including chitinases, together with extracellular carbohydrate-active enzymes such as pectinases (Földi et al. 2024).

### Core co-expression network and Zn(II)_2_Cys_6_ transcription factors

Weighted gene co-expression network analysis (WGCNA) identified the MEturquoise module as being positively associated with reproductive development, with the strongest positive correlation observed for mature fruiting bodies and a moderate correlation with primordia. In addition to structural proteins, highly connected genes within this module included predicted transcription factors (TFs), chromatin-associated regulators, and components of the ubiquitin–proteasome system. This network composition suggests that coordinated transcriptional regulation, chromatin modification, and protein turnover may contribute to the developmental transition of *A. ostoyae*. Notably, the promoter regions of 12 predicted Zn(II)_2_Cys_6_ transcription factor genes contained low-temperature-responsive elements (LTREs). The core CCGAC sequence of the LTRE has been associated with cold-responsive gene regulation in plants (Hao et al. 2002; Zolotarov and Strömvik 2015). Although the functional significance of these predicted elements in *A. ostoyae* remains to be experimentally determined, their presence suggests that some AoZCy6 genes may participate in low-temperature-associated transcriptional responses.

To further evaluate the expression patterns supporting the transcriptomic model, we performed quantitative real-time PCR (qRT-PCR) profiling of selected candidate genes, including *AoZCy6_17*, across vegetative mycelia, primordia, and mature fruiting bodies. Several candidates showed pronounced stage-associated increases in expression in primordia and/or mature fruiting bodies, broadly supporting the developmental expression patterns detected by RNA-seq (Suppl. material 2: fig. S5; Suppl. material 1: table S21). These observations are consistent with the potential involvement of AoZCy6 factors in early reproductive development, although they do not establish direct cold sensing or regulatory relationships. Based on mechanisms described in other fungi, cold exposure may initially alter membrane properties and activate membrane-associated histidine kinases, calcium channels, or related signaling components (Tai et al. 2007). The resulting signaling pathways may subsequently influence the expression of LTRE-containing AoZCy6 genes, which could contribute to downstream transcriptional regulation; however, this proposed regulatory sequence remains hypothetical.

Predicted candidate targets associated with these Zn(II)_2_Cys_6_ factors included glycoside hydrolases such as GH47, glycosyltransferases such as GT8, and antioxidant enzymes, including superoxide dismutase (SOD) and catalase (CAT). These functional categories are consistent with processes required during primordium development: glycoside hydrolases and glycosyltransferases may contribute to cell-wall remodeling, whereas SOD and CAT may help regulate reactive oxygen species generated during low-temperature exposure and developmental growth (Nagy et al. 2023). Functional analysis of *AoZCy6_17* further showed that its overexpression was associated with significantly elevated basal CAT activity under non-cold conditions. This result provides experimental evidence that altered *AoZCy6_17* expression influences CAT-associated oxidative physiology, but it does not by itself demonstrate that *AoZCy6_17* directly regulates CAT genes or controls the broader ROS-scavenging network.

### Potential roles of epigenetic regulation and protein homeostasis in the co-expression network

The transition from vegetative to reproductive growth requires not only the activation of reproductive-development programs but also the attenuation of vegetative-growth programs. Our data suggest the potential involvement of SET-domain methyltransferases and F-box proteins in these processes.

RNA-seq analysis revealed that several genes encoding predicted SET-domain methyltransferases were significantly upregulated during the primordium and fruiting body stages, suggesting that chromatin-state regulation may contribute to developmental transcriptional reprograming. Studies in other fungi have shown that SET-domain methyltransferases, such as DIM-5 in *Neurospora*, establish histone methylation marks that influence heterochromatin formation and gene expression, whereas other chromatin-associated regulators, including LaeA in *Aspergillus*, also contribute to fungal development and metabolism. Based on these functional precedents, the SET-domain candidates identified here may participate in modulating chromatin states during reproductive development. However, their specific histone targets and whether they promote transcriptional activation or repression in *A. ostoyae* remain to be determined (Freitag 2017; Nowrousian 2022; Zhang and Tao 2025).

Several F-box proteins were also identified among the highly connected genes in the MEturquoise module. As substrate-recognition components of Skp1–Cullin–F-box (SCF) ubiquitin ligase complexes (Gagne et al. 2002; Xu et al. 2021), F-box proteins mediate selective protein turnover and have been implicated in fungal development and environmental responses. Their presence in the module suggests that ubiquitin-mediated proteolysis may contribute to the removal or modulation of regulatory proteins during low-temperature-associated reproductive development. Low-temperature exposure may influence the expression or activity of specific F-box proteins and thereby alter the stability of developmental regulators; however, the relevant protein substrates and causal relationships remain unknown (Bayram and Braus 2012; Krizsán et al. 2019).

Preliminary attempts to examine the temporal expression profiles of selected SET-domain and F-box candidates during low-temperature induction using conventional qPCR were limited by their very low transcript abundance. Their Ct values frequently approached the upper limit of reliable quantification, preventing robust estimation of relative fold changes. Consequently, the temporal responses of these candidates remain unresolved. Further validation may require optimized primer sets, increased template input, or more sensitive quantitative approaches such as droplet digital PCR (ddPCR).

### Potential roles of hydrophobins during cryometamorphosis

Hydrophobins may contribute not only to structural differentiation but also to changes in surface properties during cryometamorphosis (Song et al. 2023). These small amphipathic proteins can self-assemble at hydrophilic–hydrophobic interfaces, reduce surface tension, and form coatings on aerial hyphae (Nagy et al. 2023). In our transcriptomic data, several hydrophobin genes showed exceptionally strong upregulation during the primordium stage, with log_2_ fold-change values exceeding 18, indicating their potential involvement in the transition from substrate-associated hyphae to aerial reproductive structures.

Recent observations have shown that fungal tissues can maintain temperatures below the surrounding environment, at least partly through evaporative cooling (Cordero et al. 2023). In combination with our transcriptomic results, these findings suggest two potential roles for hydrophobins in *A. ostoyae*:

Structural adaptation: Hydrophobins may reduce surface tension at the substrate–air interface, facilitate the emergence of aerial hyphae, and help maintain appropriate surface hydrophobicity during primordium formation (Bayry et al. 2012).

Surface moisture and thermal regulation: By modifying the hydrophilic–hydrophobic properties of the primordium surface, hydrophobins may influence water retention, droplet formation, and evaporation. These effects could contribute to the local moisture and temperature conditions experienced by developing tissues. However, whether hydrophobin-mediated surface organization contributes directly to evaporative cooling in *A. ostoyae* remains to be experimentally tested.

The *AoZCy6_17*-overexpressing line also exhibited conspicuous surface condensation and increased aerial mycelial growth at 25 °C. This phenotype indicates that *AoZCy6_17* overexpression alters colony surface architecture and moisture distribution. Although these observations are consistent with changes in surface-associated proteins or extracellular matrix properties, the present data do not establish that hydrophobins mediate this phenotype or that the accumulated droplets reflect enhanced evaporative cooling. Direct measurement of hydrophobin expression in the overexpression strain, together with surface hydrophobicity, evaporation rate, and tissue-temperature measurements, will be required to evaluate this proposed relationship.

### Functional implications of *AoZCy6_17* overexpression for morphogenesis and oxidative physiology

Although the Zn(II)_2_Cys_6_ (C6) transcription factor family is well characterized in ascomycetes and yeasts as an important regulator of fungal development, carbon metabolism, and redox homeostasis (Chang and Ehrlich 2013), its functional roles in the complex multicellular development of basidiomycetes remain less well understood. The generation of an *AoZCy6_17*-overexpressing strain in this study provides functional evidence that elevated *AoZCy6_17* expression is associated with morphological and biochemical changes relevant to early cryometamorphosis in *Armillaria ostoyae*.

The most conspicuous macroscopic phenotype was the absence of visible exploratory rhizomorph networks, accompanied by increased dense aerial mycelial growth at 25 °C. In the ecology of *Armillaria*, rhizomorphs function as exploratory and resource-translocating structures that facilitate vegetative spread. Previous genomic and transcriptomic analyses showed that rhizomorphs and fruiting bodies share subsets of highly expressed genes and promoter-associated regulatory signatures, suggesting partially overlapping morphogenetic programs (Sipos et al. 2017). Our observation that *AoZCy6_17* overexpression altered the relative development of rhizomorphs and aerial mycelia is consistent with the involvement of this transcription factor in colony-level morphological regulation. However, this phenotype does not by itself demonstrate that *AoZCy6_17* acts as a directional developmental switch or directly controls the shared regulatory machinery of rhizomorph and fruiting body development. A related Zn(II)_2_Cys_6_ transcription factor, PpZCP11, was recently shown to promote primordium formation and influence cell-wall-related gene expression in *Pleurotus pulmonarius*, further supporting the involvement of C6 transcription factors in basidiomycete reproductive morphogenesis (Wang et al. 2026).

The morphological changes associated with *AoZCy6_17* overexpression were accompanied by altered oxidative-status indicators. The significantly elevated basal CAT activity in the OE strain at 25 °C suggests enhanced catalase-associated antioxidant capacity in the absence of low-temperature treatment. By contrast, the concurrent increase in MDA content indicates greater lipid peroxidation, suggesting that elevated CAT activity did not prevent the development of an altered oxidative state. These observations are broadly consistent with findings for the Zn(II)_2_Cys_6_ transcription factor HADA-1 in *Hypsizygus marmoreus*, whose silencing affected mitochondrial stability, ROS levels, oxidative-stress sensitivity, and fruiting body development (Zhang et al. 2021). Together, the elevated CAT activity and MDA accumulation observed in the OE strain at 25 °C indicate that *AoZCy6_17* overexpression modifies oxidative physiology even in the absence of immediate cold exposure. The fact that some biochemical changes in the OE strain were comparable to or greater than those observed in the low-temperature-treated WT strain supports the importance of *AoZCy6_17* as a candidate regulatory component of the cryometamorphosis-associated response. Nevertheless, these results do not establish its position at the apex of a hierarchical regulatory cascade or demonstrate an evolutionary trade-off between metabolic efficiency and winter preparedness. Rather, they link *AoZCy6_17* expression with coordinated morphological and oxidative changes within the cryometamorphosis expression patterns associated with fruiting body development in *A. ostoyae*.

## Limitations and future perspectives

Although the establishment of an *Agrobacterium tumefaciens*-mediated transformation (ATMT) system and the generation of an *AoZCy6_17*-overexpressing strain provide functional evidence for the involvement of this candidate regulator in *A. ostoyae* development, the direct regulatory relationships within the proposed network remain to be resolved. Building on the genetic transformation system established here, future studies should employ targeted gene disruption or CRISPR–Cas9-mediated knockout, together with genetic complementation, to determine whether *AoZCy6_17* is required for the observed developmental and physiological responses. In addition, chromatin immunoprecipitation sequencing (ChIP-seq) or related chromatin-profiling approaches could be used to identify genomic regions occupied by *AoZCy6_17* and to characterize developmental changes in histone modifications associated with SET-domain proteins during low-temperature induction. Integrating these analyses with promoter-reporter assays and functional characterization of candidate downstream genes will help distinguish direct regulatory interactions from co-expression relationships and may ultimately provide molecular targets for improving the artificial cultivation of this species.

## Conclusions

This study establishes an integrated physiological, transcriptomic, and functional framework for investigating the cryometamorphosis expression patterns associated with fruiting body development in *Armillaria ostoyae*. Based on the cultivation responses observed under the conditions tested, *A. ostoyae* can be categorized as a “cold-pressed” species in which low-temperature exposure strongly promotes the transition from vegetative growth to primordium formation. The combined transcriptomic and co-expression analyses support a working model in which low-temperature-associated signaling is accompanied by the expression of selected Zn(II)_2_Cys_6_ transcription factors containing predicted LTREs, together with changes in SET-domain methyltransferases, F-box proteins, and genes involved in cell-wall remodeling, oxidative responses, and surface differentiation. Within this framework, hydrophobins may contribute to aerial hyphal development and changes in colony-surface properties, although their proposed thermoregulatory role remains hypothetical.

The establishment of genetic transformation and the overexpression of the core candidate regulator *AoZCy6_17* provided functional support for its involvement in this developmental response. Elevated *AoZCy6_17* expression was associated with the absence of visible rhizomorph formation, increased aerial mycelial growth, and altered CAT activity and MDA accumulation. These findings do not establish *AoZCy6_17* as a master regulator or define direct downstream targets, but they link its expression to coordinated morphological and oxidative changes relevant to cryometamorphosis. Overall, this study provides a testable molecular framework for understanding how low-temperature cues are associated with transcriptional reprograming and macroscopic developmental changes in *A. ostoyae*.

## Additional information

### Artificial Intelligence (AI) use

The authors accept full responsibility for the content of the manuscript, including the disclosure of any use of AI.

The following AI tools were used in the preparation of this manuscript:

**AI tools**: Gemini, GPT.

**Used for**: Language editing, style and writing, data analysis.

**Description**: Gemini and GPT were used to assist with language editing, style and writing, data analysis, and manuscript consistency checking. All scientific content, analyses, interpretations, and final wording were reviewed and approved by the authors.

### Adherence to national and international regulations

All the fungal strains used in this study have been legally obtained, respecting the Convention on Biological Diversity (Rio Convention).

### Author ORCIDs

Z.-X. Zhu https://orcid.org/0000-0002-8330-918X

### Data availability

The raw RNA-sequencing data generated in this study have been submitted to the Genome Sequence Archive (GSA) at the National Genomics Data Center, China National Center for Bioinformation / Beijing Institute of Genomics, Chinese Academy of Sciences, under accession number CRA036414, within BioProject PRJCA055014. The reference genome of Armillaria ostoyae used for alignment is available at NCBI under accession number GCA_900157425.1 (Sipos et al. 2017). All other data supporting the findings of this study are available within the article and Supplementary information.

## Supplementary materials

Supplementary material 1 Supplementary tables Data type xlsx https://binary.pensoft.net/file/1738571This dataset is made available under the Open Database License (http://opendatacommons.org/licenses/odbl/1.0/). The Open Database License (ODbL) is a license agreement intended to allow users to freely share, modify, and use this Dataset while maintaining this same freedom for others, provided that the original source and author(s) are credited. Ji-Jiang Zhou, Yi-Jing Zhao, Qi Liu, Jin Liu, Si-Hong Li, Zhong-Yi Wang, Zhao-Xiang Zhu, Xin-Shuai Xiong, Rui-Qing Ji

Supplementary material 2 Supplementary figures Data type pdf https://binary.pensoft.net/file/1738572This dataset is made available under the Open Database License (http://opendatacommons.org/licenses/odbl/1.0/). The Open Database License (ODbL) is a license agreement intended to allow users to freely share, modify, and use this Dataset while maintaining this same freedom for others, provided that the original source and author(s) are credited. Ji-Jiang Zhou, Yi-Jing Zhao, Qi Liu, Jin Liu, Si-Hong Li, Zhong-Yi Wang, Zhao-Xiang Zhu, Xin-Shuai Xiong, Rui-Qing Ji
