# Supplementary material for: Cryometamorphosis expression patterns of fruiting body development in the mushroom-forming fungus Armillaria ostoyae
Source: IMA Fungus. 2026 Aug 6;17:e186942. doi: 10.3897/imafungus.17.186942 (PMC13473897; doi:10.3897/imafungus.17.186942)
Supplement: Supplementary file 1 [file imafungus-17-e186942-s002.pdf]

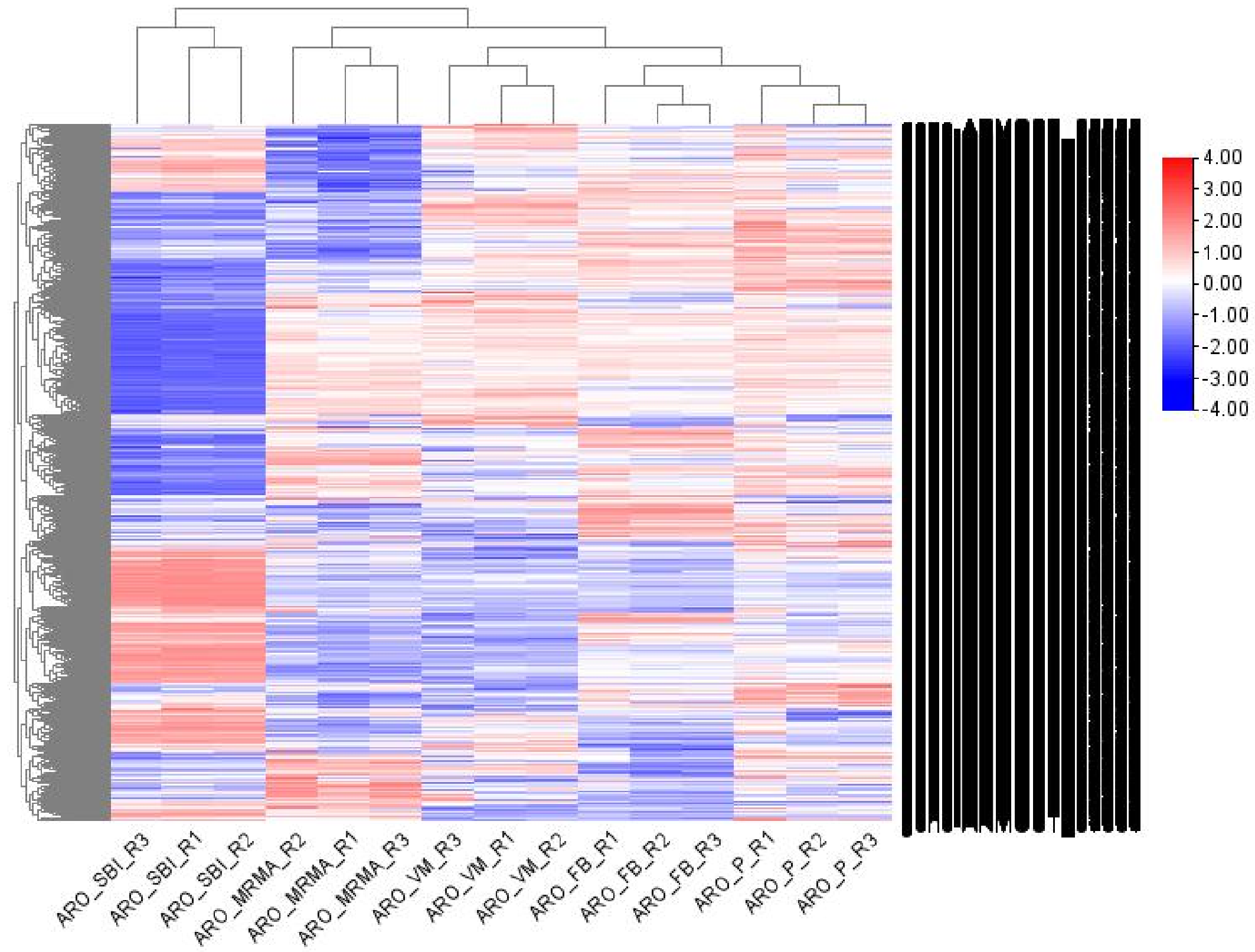

Supplementary Figure 1. Global gene expression patterns across developmental and morphological states of *Armillaria ostoyae* JG19016 inferred from RNA-seq data. Three independent biological replicates were analyzed for each state. Abbreviations are as follows: ARO_MRM, melanized rhizomorph; ARO_VM, vegetative mycelium; ARO_FB, mature fruiting body; ARO_P, primordium; and ARO_SBI, monokaryotic mycelium derived from a single-spore isolate.

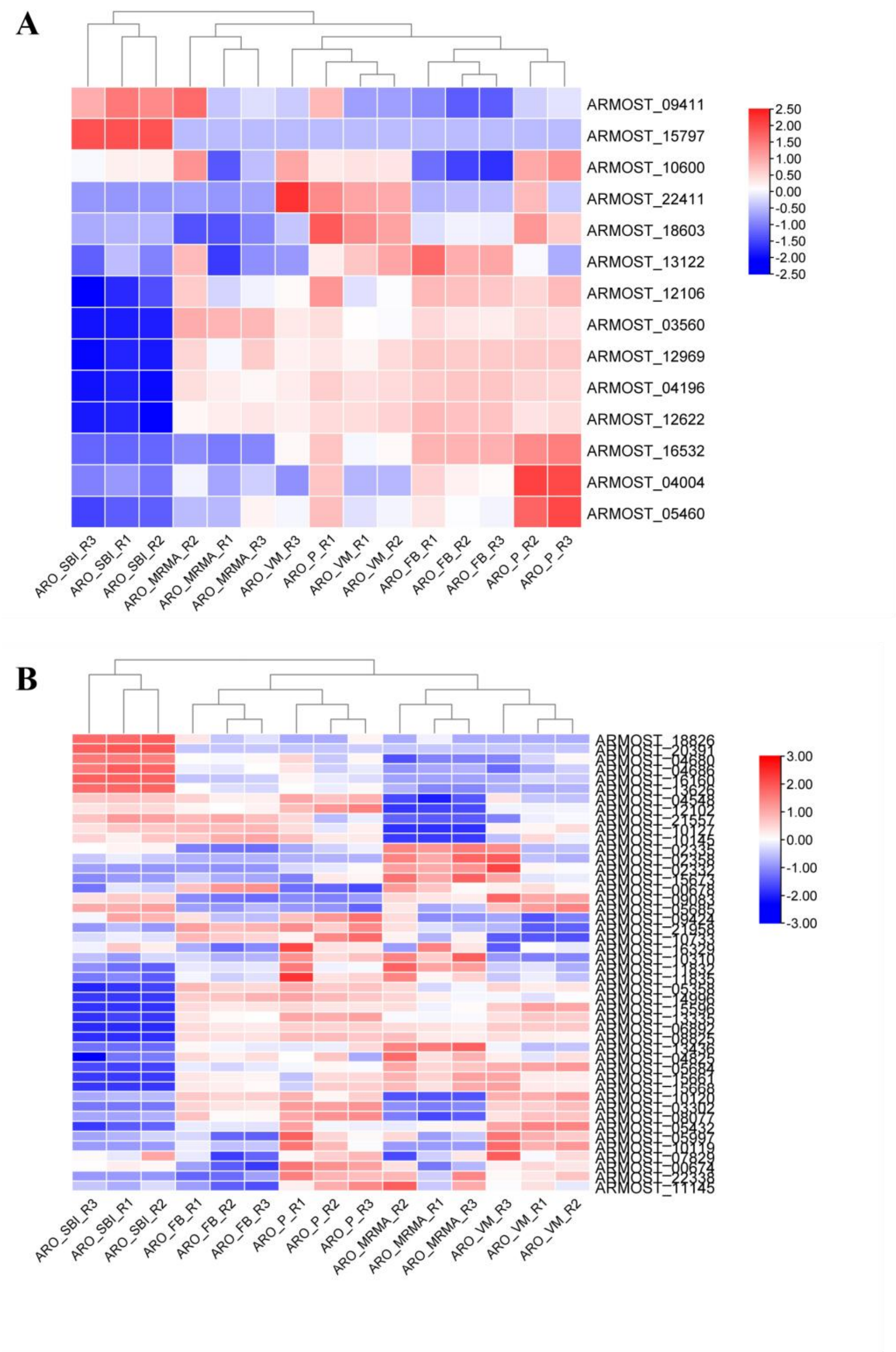

Supplementary Figure 2. Expression patterns of genes associated with putative stimulus-responsive pathways in *Armillaria ostoyae* JG19016. Heatmaps show the expression profiles of genes encoding fatty acid desaturases and phospholipases (A) and genes associated with calcium signaling (B) across developmental and morphological states. Abbreviations are as follows: ARO_MRM, melanized rhizomorph; ARO_VM, vegetative mycelium; ARO_FB, mature fruiting body; ARO_P, primordium; and ARO_SBI, monokaryotic mycelium derived from a single-spore isolate.

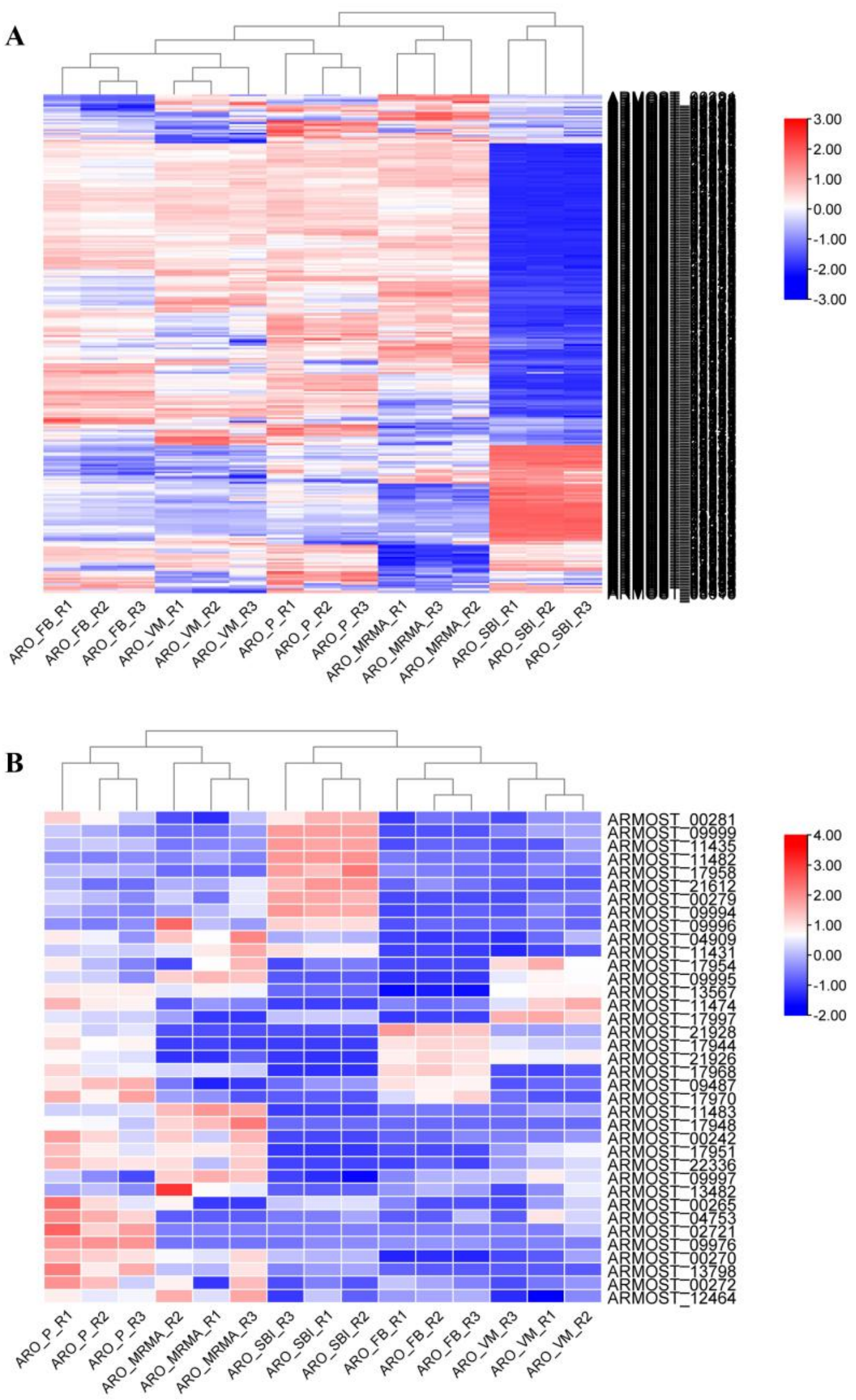

Supplementary Figure 3. Expression patterns of predicted transcription factor and hydrophobin genes in *Armillaria ostoyae* JG19016. Heatmaps show the expression profiles of differentially expressed genes encoding predicted transcription factors (A) and hydrophobins (B) across developmental and morphological states. The primordium stage contained the largest number of upregulated predicted transcription factor genes, including several with high expression levels. A distinct subset of predicted transcription factor genes was preferentially expressed in the monokaryotic mycelial state. Hydrophobin genes showed a partially overlapping expression pattern in the primordium and monokaryotic mycelial states. Abbreviations are as follows: ARO_MRM, melanized rhizomorph; ARO_VM, vegetative mycelium; ARO_FB, mature fruiting body; ARO_P, primordium; and ARO_SBI, monokaryotic mycelium derived from a single-spore isolate.

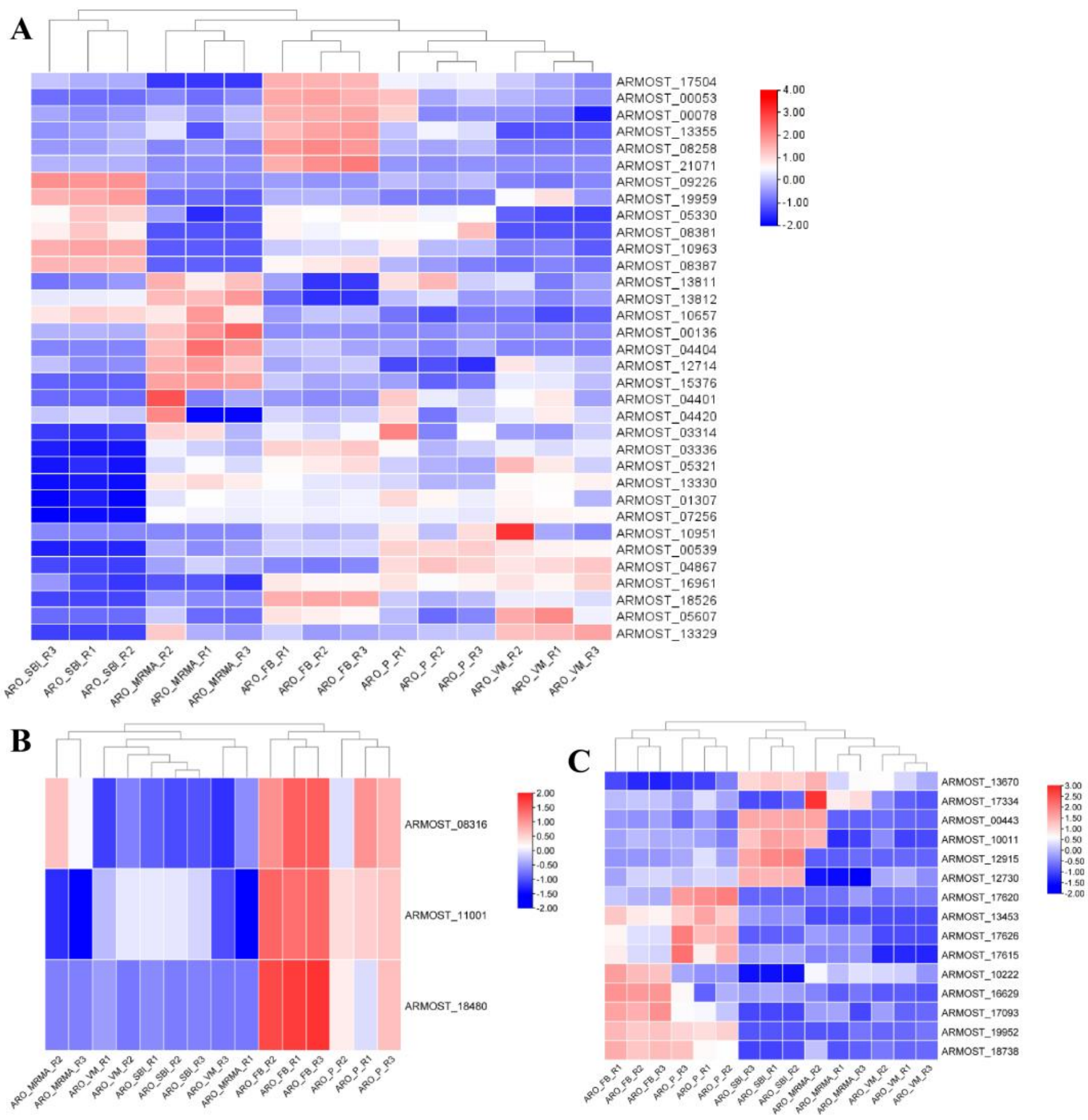

Supplementary Figure 4. Expression patterns of cell wall biosynthesis, pore-forming toxin, and GMC oxidoreductase genes in *Armillaria ostoyae* JG19016. Heatmap visualization of differentially expressed genes involved in (A) cell wall biosynthesis, (B) pore-forming toxins, and (C) GMC oxidoreductases across developmental stages. Expression clustering of these three morphogenesis-related gene groups distinctly separates melanized rhizomorphs and monokaryotic isolates from other developmental stages. The monokaryotic stage shows high expression of a specific subset of GMC oxidoreductases. Abbreviations are as follows: ARO_MRM – melanized rhizomorph, ARO_VM – vegetative mycelium, ARO_FB – fruiting body, ARO_P – primordium , ARO_SBI – single spore isolate/ monokaryon.

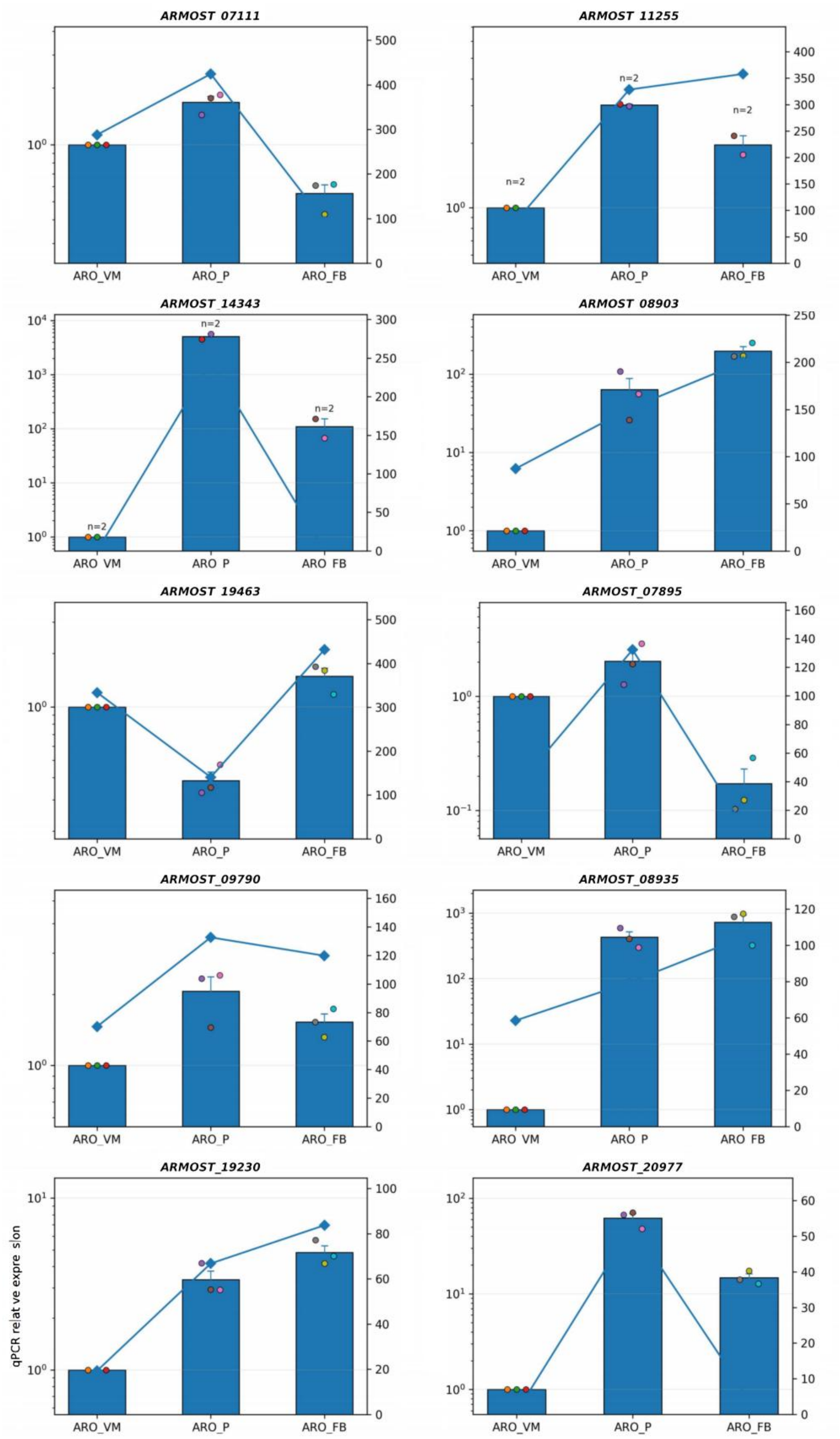

Supplementary Figure 5. Comparison of qPCR and RNA-seq expression profiles of ten representative ARMOST candidate genes across developmental stages. Bars represent the mean relative expression, error bars indicate the SEM, and individual points represent matched biological replicates. Red lines and diamond markers show the mean RNA-seq expression levels in transcripts per million (TPM). The left y-axis presents qPCR relative expression on a base-10 logarithmic scale, whereas the right y-axis presents RNA-seq expression on a linear scale. ARO_VM was fixed at 1 by definition. Effective replicate numbers were n=3 for eight genes and n=2 for ARMOST_11255 and ARMOST_14343 after technical-replicate quality control.

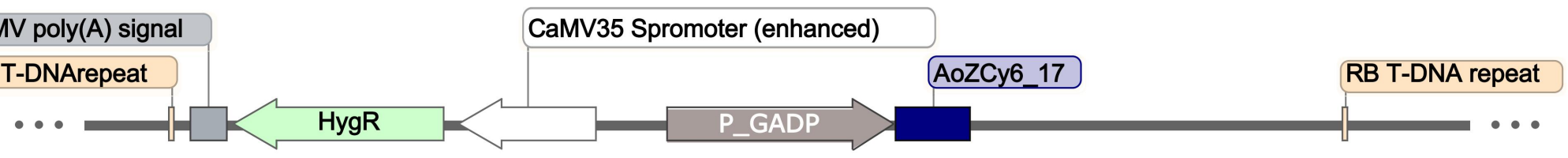

Supplementary Figure S6. Schematic representation of the T-DNA region of the AoZCy6_17 overexpression construct.
